# Pulse wave velocity and central aortic pressure in systolic blood pressure intervention trial participants

**DOI:** 10.1371/journal.pone.0203305

**Published:** 2018-09-26

**Authors:** Mark A. Supiano, Laura Lovato, Walter T. Ambrosius, Jeffrey Bates, Srinivasan Beddhu, Paul Drawz, Jamie P. Dwyer, Naomi M. Hamburg, Dalane Kitzman, James Lash, Eva Lustigova, Cynthia M. Miracle, Suzanne Oparil, Dominic S. Raj, Daniel E. Weiner, Addison Taylor, Joseph A. Vita, Reem Yunis, Glenn M. Chertow, Michel Chonchol

**Affiliations:** 1 Geriatrics Division University of Utah School of Medicine and VA Salt Lake City Geriatric Research, Education and Clinical Center, Salt Lake City, Utah, United States of America; 2 Department of Biostatistical Sciences, Wake Forest School of Medicine, Winston Salem, North Carolina, United States of America; 3 Michael E. DeBakey VA Medical Center and Baylor College of Medicine, Houston, Texas, United States of America; 4 Nephrology Division University of Utah and Salt Lake City VA Medical Center, Salt Lake City, Utah, United States of America; 5 Division of Renal Diseases & Hypertension, University of Minnesota, Minneapolis, Minnesota, United States of America; 6 Division of Nephrology/Hypertension, Vanderbilt University Medical Center, Nashville, Tennessee, United States of America; 7 Section of Vascular Biology, Boston University School of Medicine, Boston, Massachusetts, United States of America; 8 Sections on Cardiovascular Medicine and Geriatrics, Wake Forest School of Medicine, Winston Salem, North Carolina, United States of America; 9 Section of Nephrology, University of Illinois at Chicago, Chicago, Illinois, United States of America; 10 Tulane University School of Public Health and Tropical Medicine, New Orleans, Louisiana, United States of America; 11 Division of Nephrology and Hypertension, University of California San Diego and VA San Diego Healthcare System, San Diego, California, United States of America; 12 Vascular Biology and Hypertension Program, Division of Cardiovascular Disease, Department of Medicine, School of Medicine, The University of Alabama at Birmingham, Birmingham, Alabama, United States of America; 13 Division of Kidney Diseases and Hypertension, George Washington University, Washington, DC, United States of America; 14 Division of Nephrology, Tufts Medical Center, Boston, Massachusetts, United States of America; 15 Division of Nephrology, Stanford University School of Medicine, Palo Alto, California, United States of America; 16 Division of Renal Diseases and Hypertension, University of Colorado Anschutz Medical Campus, Denver, Colorado, United States of America; Shanghai Institute of Hypertension, CHINA

## Abstract

Arterial stiffness, typically assessed as the aortic pulse wave velocity (PWV), and central blood pressure levels may be indicators of cardiovascular disease (CVD) risk. This ancillary study to the Systolic Blood Pressure Intervention Trial (SPRINT) obtained baseline assessments (at randomization) of PWV and central systolic blood pressure (C-SBP) to: 1) characterize these vascular measurements in the SPRINT cohort, and 2) test the hypotheses that PWV and C-SBP are associated with glucose homeostasis and markers of chronic kidney disease (CKD). The SphygmoCor^®^ CPV device was used to assess carotid-femoral PWV and its pulse wave analysis study protocol was used to obtain C-SBP. Valid results were obtained from 652 participants. Mean (±SD) PWV and C-SBP for the SPRINT cohort were 10.7 ± 2.7 m/s and 132.0 ± 17.9 mm Hg respectively. Linear regression analyses for PWV and C-SBP results adjusted for age, sex, and race/ethnicity in relation to several markers of glucose homeostasis and CKD did not identify any significant associations with the exception of a marginally statistically significant and modest association between PWV and urine albumin-to-creatinine ratio (linear regression estimate ± SE, 0.001 ± 0.0006; P-value 0.046). In a subset of SPRINT participants, PWV was significantly higher than in prior studies of normotensive persons, as expected. For older age groups in the SPRINT cohort (age > 60 years), PWV was compared with a reference population of hypertensive individuals. There were no compelling associations noted between PWV or C-SBP and markers of glucose homeostasis or CKD.

**Clinical Trial Registration**: NCT01206062.

## Introduction

Arterial stiffness–typically assessed as central (aortic) carotid-femoral pulse wave velocity (PWV)–is an indicator of cardiovascular disease (CVD) risk independent of both the peripheral (brachial) BP and pulse pressure.[1–3] Recent meta-analyses have identified PWV as a predictor of future CVD events and all-cause mortality independent of blood pressure.[4, 5] Additional studies have demonstrated an association between PWV and subsequent declines in both kidney function and cognitive function.[6–11] Furthermore, many studies have demonstrated that measures of central aortic blood pressure are associated with CV outcomes and mortality independent of peripheral brachial arterial pressure, and may provide a better measure to guide antihypertensive therapy. [12]

The Systolic Blood Pressure Intervention Trial (SPRINT, clinicaltrials.gov: NCT01206062) was designed to test the hypothesis that treatment to a more intensive systolic blood pressure (SBP) target of < 120 mm Hg would reduce cardiovascular morbidity and mortality versus a standard SBP target of <140 mm Hg among non-diabetic adults.[13] This SPRINT ancillary study obtained baseline assessments of vascular stiffness and central aortic pressure in a subset of participants at the time of their randomization into the SPRINT study. These baseline results are presented to address two objectives: 1) to characterize measures of vascular stiffness and central pressure in persons meeting the SPRINT inclusion criteria[13], and 2) to test the hypotheses that vascular stiffness and central pressure are associated with markers of glucose homeostasis (fasting glucose, insulin, hemoglobin A_1_C) renin, aldosterone, and markers associated with CKD (serum calcium, phosphorous, parathyroid hormone, uric acid, and urine albumin-to-creatinine ratio (UACR)).

## Methods

### Study cohort and clinical study sites

**Fig 1:**
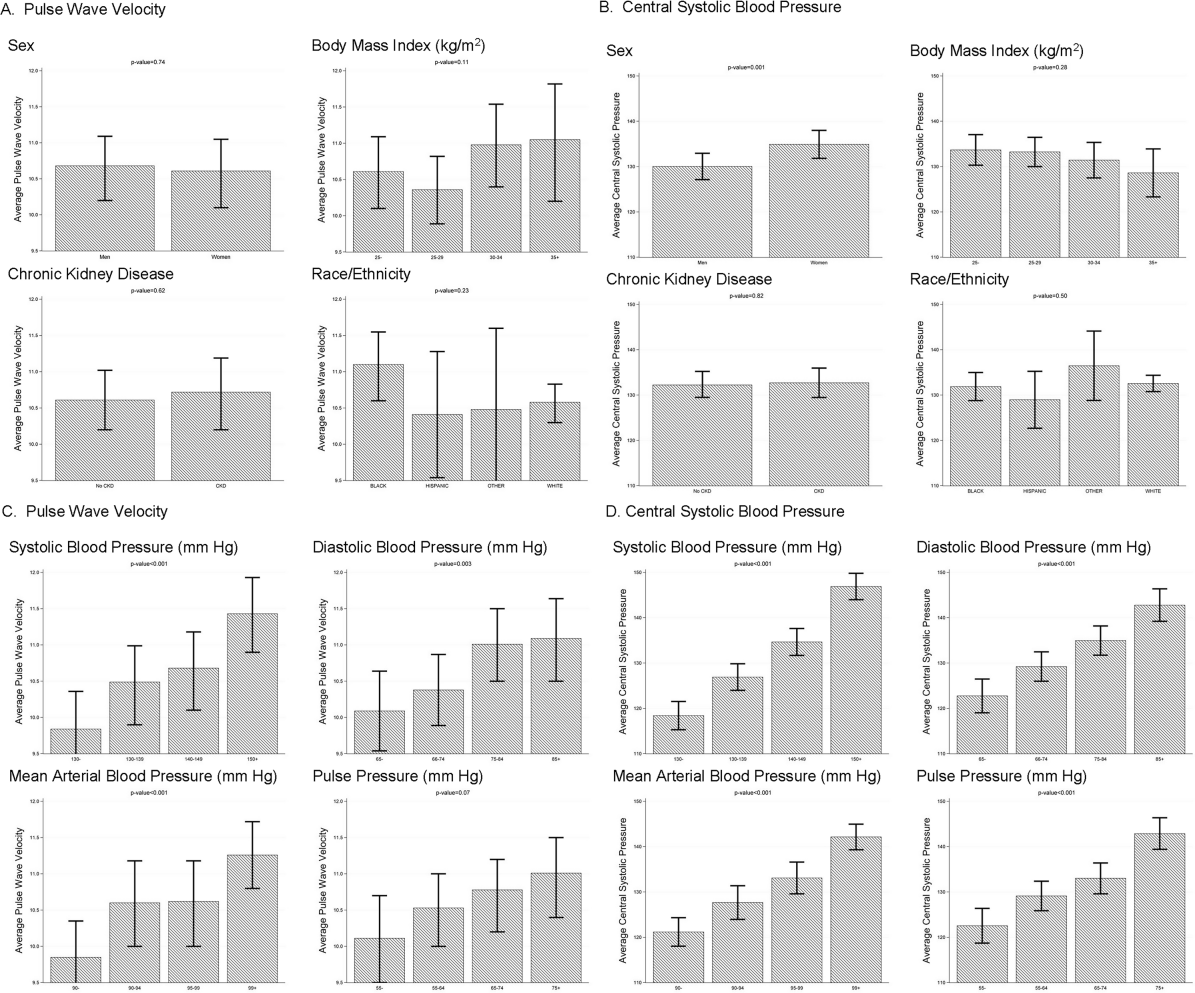
Least square means and 95% confidence intervals for pulse wave velocity and central aortic blood pressure: generalized linear models with adjustments for sex, race, and age.

The design of the SPRINT study has been reported.[13] Briefly, 9,361 participants were randomized to either a standard SBP target of <140 mm Hg or a more intensive SBP target of < 120 mm Hg. Participants were eligible for SPRINT if they were at least 50 years of age and had a SBP at a screening visit within the range of 130–180, 130–170, 130–160, or 130–150 mm Hg while being on no more than 0 or 1, 2, 3, or 4 anti-hypertensive medications, respectively. SPRINT specifically targeted high-risk participants, including those with prior CVD, chronic kidney disease (CKD), defined as an estimated glomerular filtration rate (eGFR) between 20 and 60 ml/min/1.73 m^2^, and persons age 75 years and older. For participants younger than 75 years old, either pre-existing CVD, CKD, or a Framingham risk score calculated to be above 15% was required. Notable exclusions included a history of diabetes mellitus, stroke, congestive heart failure. Additional exclusion criteria for the Senior subgroup included: a clinical diagnosis of or treatment for dementia, an expected survival of less than 3 years, unintentional weight loss (>10% of body weight) during the preceding 6 months, an SBP of less than 110 mm Hg following 1 minute of standing, or residing in a nursing home. The study interventions ended earlier than planned when the Data Safety Monitoring Board (DSMB) noted that the primary study outcome–a composite CVD outcome comprised of myocardial infarction, acute coronary syndrome, stroke, heart failure, or CVD death–and all-cause mortality were significantly lower in the intensive blood pressure treatment group.[14] The peripheral brachial blood pressures reported here (Tables 1 and 2 and Fig 1) represent the participants’ seated BP measured using the SPRINT measurement protocol at the time of randomization. [13]

**Table 1:** Baseline characteristics comparing SPRINT participants in PWV ancillary study to the entire cohort.

		PWV Ancillary Study		
	Overall (N = 9361)	Yes (N = 652)	No (N = 8709)	P-value
Age (years; mean ± SD)	67.9 ± 9.4	72.2 ± 9.3	67.6 ± 9.4	<0.0001
Age in years				<0.0001
50–59	1963 (21.0%)	84 (12.9%)	1879 (21.6%)	
60–69	3409 (36.4%)	131 (20.1%)	3278 (37.6%)	
70–79	2822 (30.1%)	285 (43.7%)	2537 (29.1%)	
> 80	1167 (12.5%)	152 (23.3%)	1015 (11.7%)	
Female	3332 (35.6%)	265 (40.6%)	3067 (35.2%)	0.005
Race/Ethnicity				<0.0001
African American	2802 (29.9%)	146 (22.4%)	2656 (30.5%)	
Hispanic	984 (10.5%)	35 (5.4%)	949 (10.9%)	
Other	176 (1.9%)	21 (3.2%)	155 (1.8%)	
White	5399 (57.7%)	450 (69.0%)	4949 (56.8%)	
Clinical or Subclinical CVD *	1877 (20.1%)	85 (13.0%)	1792 (20.6%)	<0.0001
Number of CVD Risk Factors *				<0.0001
None	7484 (79.9%)	567 (87.0%)	6917 (79.4%)	
One	1009 (10.8%)	54 (8.3%)	955 (11.0%)	
Two	503 (5.4%)	17 (2.6%)	486 (5.6%)	
Three	244 (2.6%)	7 (1.1%)	237 (2.7%)	
Four or More	121 (1.3%)	7 (1.1%)	114 (1.3%)	
Current Smoker	1240 (13.3%)	44 (6.8%)	1196 (13.8%)	<0.0001
Systolic BP (mm Hg)	139.7 ± 15.6	140.5 ± 15.2	139.6 ± 15.6	0.16
Diastolic BP (mm Hg)	78.1 ± 11.9	75.1 ± 12.0	78.4 ± 11.9	<0.0001
Heart Rate (bpm)	66.3 ± 11.6	64.7 ± 10.9	66.4 ± 11.6	0.0005
Weight (kg)	86.6 ± 18.8	80.4 ± 16.8	87.1 ± 18.9	<0.0001
BMI (kg/m^2^)	29.9 ± 5.8	27.9 ± 5.0	30.0 ± 5.8	<0.0001
Assigned to Intensive Arm	4678 (50.0%)	320 (49.1%)	4358 (50.0%)	0.64
In Senior Subgroup	2636 (28.2%)	311 (47.7%)	2325 (26.7%)	<0.0001
In CKD Subgroup	2645 (28.4%)	229 (35.2%)	2416 (27.9%)	<0.0001

* Detailed definitions of the CVD risk factors are provided in reference [13]

**Table 2:** Pulse wave velocity and systolic blood pressures by age group in SPRINT cohort and reference populations with grade i hypertension (≥140/90 and ≤160/100 mm Hg)[17].

	SPRINT Participants			Reference Values for Arterial Stiffness Collaboration with Grade I Hypertension	
Age (years)	n	Systolic Blood Pressure (mm Hg)	PWV (m/s)	n	PWV (m/s)
50–59	84	137 (17)	9.6 (5.3–13.9)	490	9.6 (4.9–14.3)
60–69	131	137 (13)	9.8 (4.0–15.6)*	648	11.1 (6.1–16.2)
≥ 70	437	142 (15)	11.2 (5.8–16.5)*	535	12.9 (6.9–18.9)

Blood pressure values are means (SD). PWV values are means ± 2 SD

* P < 0.001 relative to Grade I Hypertension

Participants enrolled in this SPRINT ancillary study were recruited from 11 of its 102 clinical sites– 10 from a single clinical center network (University of Chicago, George Washington University, University of Texas Southwestern, University of Colorado, University of Utah, University of Pittsburgh, Tufts Medical Center, Vanderbilt University, Stanford University, and University of California-San Diego) plus the Houston VA Medical Center. The study protocol was approved by Institutional Review Boards at each of the sites–Stanford University Institutional Review Board, Tufts Health Sciences Institutional Review Board, Colorado Multiple Institutional Review Board, University of Illinois at Chicago Institutional Review Board, University of Pittsburgh Institutional Review Board, University of Texas Southwestern Medical Center Institutional Review Board, University of Utah Institutional Review Board, Vanderbilt University Institutional Review Board, The George Washington University Institutional Review Board, Veterans Affairs San Diego Healthcare System Institutional Review Board, and the Michael E. DeBakey VA Medical Center Institutional Review Board–and all participants provided written informed consent. The only additional exclusion criterion for entry into the ancillary study was the presence of atrial fibrillation, since this arrhythmia precludes obtaining a valid PWV result using the SphygmoCor^®^ device. Seven hundred and seventy-one SPRINT participants agreed and signed informed consent for the ancillary study. Consistent with a projected 4% ineligible rate (due to atrial arrhythmias) and 5–13% inadequate study rate, valid PWV data were obtained from 652 participants at baseline.

### Study protocol

AtCor Medical provided each study site with the SphygmoCor CPV system device with software version 9.0 dedicated solely for use in this study. AtCor Medical trainers visited each site to train study personnel in proper performance of the PWV and pulse wave analysis (PWA) study protocols as detailed in a study manual of procedures.

The study protocol coincided with participants’ SPRINT randomization study visit. In some instances when this schedule could not be kept, a separate visit was scheduled within 1 month of randomization. The study’s standardized procedures adhered to the recommendations published by the Consensus Conference on the Clinical Applications of Arterial Stiffness.[15] Participants were instructed to not eat or drink other than water and to not smoke for at least 6 hours before the studies, and to not exercise after midnight prior to the studies.

The PWA and PWV protocols were then conducted. In brief, with the participant in a supine position, a hand-held high-fidelity tonometer was sequentially placed over the maximal impulse of each measurement location (radial, carotid and femoral arteries) to achieve a pressure wave contour with a consistent baseline, contour, and amplitude. The R-wave of a simultaneously-recorded ECG was used as a time base to measure pulse transit times (PTT) to each measurement location. A twenty-second time span of pulse contours (minimum of 10 pulse waves) was recorded. For PWV, a set of calipers was used to measure the distances between the participant’s carotid and femoral pulse locations and the suprasternal notch (SSN). Carotid-femoral PWV was calculated as the difference between the SSN-femoral and SSN-carotid distances divided by the difference between the PTT values for each location. The Manual of Procedures stipulated that the standard deviation of the Pulse Wave Velocity measurement needed to be less than or equal to 10%. A measurement was excluded if the pressure contour was of poor quality or if a significant difference (>5 bpm) in heart rate was found between the carotid and femoral measurements. Four PWV measurements were recorded for each participant. A participant’s PWV was the average of the technically acceptable measurements. For PWA, the Manual of Procedures stipulated that 10 radial artery pressure contour waves were obtained that were similar in amplitude with well defined feet, clean and sharp upstrokes, and signal strength ≥ to 300. If the standard deviation was less than or equal to 10% the “Operator Index” was checked to be certain it exceeded 75. If this was less than 75, the PWA procedure was to be repeated. A validated generalized transfer function was used to derive the ascending aortic pressure waveform from the non-invasively measured radial artery pressure waveform.[16] The transfer function incorporated the peripheral blood pressure determined just prior to the PWA protocol. After 10 minutes of rest in the supine position using an automated blood pressure device blood pressure was measured three times, waiting at least one minute between readings. The average of three BP readings was used together with frequency components of the peripheral waveform to synthesize the aortic waveform parameters–the central aortic SBP (C-SBP) is the maximum pressure of the aortic waveform.

To ensure standardization and quality control of the PWA and PWV measures across clinical sites, the study data files were transmitted to a Vascular Core Laboratory at Boston University for review. A single technician at the core lab reviewed each study to ensure quality control for the measures across sites. Only studies deemed to meet its quality control standards were sent to the SPRINT Coordinating Center for inclusion in the study data.

### Laboratory biomarker assessment

Blood samples submitted to the SPRINT parent study’s central lab were used to analyze several biomarkers of interest pertaining to vascular stiffness. Several of these assays–insulin, glycosylated hemoglobin, aldosterone, serum calcium and phosphorus, uric acid, and parathyroid hormone–were not available for the entire SPRINT cohort, and were performed only for participants in this ancillary study. The biochemical parameters include measures of glucose regulation (fasting glucose and insulin and glycosylated hemoglobin levels), kidney function and several markers related to CKD (eGFR, UACR, serum albumin, calcium, phosphorus, and parathyroid hormone) and levels of uric acid and aldosterone. Insulin levels were determined by a insulin reagent/sandwich immunoassay method (Roche Cobas e411 analyzer; Roche Diagnostics, Indianapolis, IN). Aldosterone levels were measured using a chemiluminescent competitive immunoassay (Diasorin, Inc, Stillwater, MN). Glycosylated hemoglobin was assayed using boronate affinity HPLC (Tosoh HPLC Glycohemoglobin Analyzer; Tosoh Medics, Inc.; San Francisco, CA). Intact parathyroid hormone was measured using a reagent/sandwich immunoassay method (Roche Diagnostics, Indianapolis, IN). The homeostasis model assessment–insulin resistance index HOMA-IR–calculated from the fasting glucose and insulin levels– [[(fasting glucose in mmol/l)*(fasting insulin in mU/ml)] / 22.5]–was used as a measure of insulin sensitivity.

### Statistical analysis

All statistical analyses were conducted at the SPRINT Coordinating Center with the use of SAS software, version 9.4 (SAS Institute, Cary, NC, USA). Continuous variables are presented as mean and standard deviation (SD) or median and 25^th^, 75^th^ percentile range, and categorical variables are presented as number with percent. Baseline characteristics were compared among participants who were or were not in this ancillary study, with the use of the chi-square test, Wilcoxon rank-sum test, and two-sample Student t-test where appropriate.

Baseline PWV in the SPRINT-PWV cohort was compared to Grade 1 Hypertension levels from reference populations reported by The Reference Values for Arterial Stiffness Collaboration[17], using t-tests within each of the three age groups. Pulse wave velocity and C-SBP were compared across sex, body mass index, peripheral SBP, mean arterial pressure, pulse pressure, existing CKD, and race/ethnicity using linear models adjusted for sex (except for the sex-stratified analysis) and race/ethnicity (except for the race/ethnicity-stratified analysis). Least squared means were computed for the categories shown in Fig 1. Similarly, we conducted linear regression analysis using either PWV or C-SBP as the dependent variable, evaluating various biomarkers after adjustment for age, sex and race/ethnicity. Pearson correlation coefficients were computed between the following five variables: PWV, peripheral SBP, C-SBP, age, and Quételet’s (body mass) index (BMI). No adjustments were made for multiple testing. Nominal P-values are reported throughout as simple guides to possible associations.

## Results

Valid PWV results could not be obtained in 123 (15.4%) participants because of either atrial arrhythmias or obesity, the latter of which precluded obtaining an accurate femoral artery wave form. Demographic and clinical characteristics for the remaining 652 participants who participated in this SPRINT ancillary study and for whom a valid PWV measure was obtained are provided in Table 1. Relative to the other 8,709 SPRINT study participants, the PWV study cohort was older, somewhat less ethnically diverse, included fewer current smokers, less likely overweight or obese, more likely to be in the CKD subgroup (eGFR 20–60 mL/min/1.73 m^2^) and less likely to be in the group with prevalent clinical or subclinical CVD. The number of CVD risk factors was lower in the PWV cohort since some participants were included because of low eGFR without other CVD risk factors. The mean (± SD) baseline PWV was 10.7 ± 2.7 m/s and C-SBP was 132.0 ± 17.9 mm Hg.

### PWV and C-SBP unadjusted correlations

As expected, there were direct correlations between PWV and age (r = 0.291; P < 0.0001), peripheral SBP (r = 0.256; P < 0.0001), and C-SBP (r = 0.204; P < 0.0001). C-SBP was weakly associated with age (r = 0.086; P = 0.03). However, there was no significant correlation between PWV and BMI (r = – 0.041; P = 0.30). There was an inverse correlation between C-SBP and BMI (r = – 0.121; P < 0.004).

### PWV by age and relative to reference groups

The age distribution of PWV results by decade are presented in Table 2. The SPRINT cohort data are shown in comparison with PWV results that have been published across a similar age range for a reference population for those with Grade I Hypertension (SBP 140 to 160 mm Hg) as defined for this referent population.[17] It should be noted that that participants taking antihypertensive drugs were excluded from this reference population study. In comparison to a Grade I Hypertension group, SPRINT PWV values were similar for the age 50–59 year group and lower for the two groups age 60 years and older.

### Multivariable analysis

Fig 1 presents least square means and 95% confidence intervals for PWV and C-SBP obtained from the adjusted generalized linear model results for demographic subgroups (sex, race/ethnicity and eGFR groups), as well as quartiles of BMI (Fig 1 Panels A and B), peripheral SBP (P-SBP), peripheral diastolic BP (DBP), mean arterial pressure and pulse pressure (Fig 1 Panels C and D). For PWV, there were no significant differences noted with respect to sex, race/ethnicity groups or presence of low eGFR. As expected, PWV remained significantly associated with peripheral SBP, DBP, mean arterial BP, and pulse pressure. Central SBP was significantly lower in men than in women (P = 0.001). A sex difference in brachial, peripheral SBP was also identified (male 138.8 ± 14.7 vs. female 143.0 ± 15.6, means ± SD; P < 0.0004). Central SBP and BMI remained significantly associated. There were no significant differences in C-SBP with respect to race/ethnicity group or eGFR subgroup. Finally, as expected, C-SBP remained significantly associated with peripheral SBP, DBP, mean arterial BP, and pulse pressure.

Table 3 presents results from linear regression analyses for PWV and C-SBP in relation to several putative biomarkers that may be related to PWV and/or C-SBP, adjusted for age, race and sex. These values are available for only participants in this ancillary study. The baseline values for these results are provided in S1 Table. The biomarkers are grouped based on their relation to glucose homeostasis (fasting glucose, insulin, HOMA-IR, and glycosylated hemoglobin levels), CKD (serum albumin, calcium, eGFR, parathyroid hormone, phosphorus, and UACR), or other (serum aldosterone and uric acid). Of these, there was a marginally statistically significant and modest association between PWV and UACR (linear regression estimate ± SE, 0.001 ± 0.0006; P-value 0.046). There was a statistically significant but relatively weak association between C-SBP and serum calcium; no other associations were identified.

**Table 3:** Linear regression analysis of various markers with PWV and central SBP, adjusted for age, race, and sex.

	PWV (m/sec)			Central SBP (mmHg)		
Marker	Estimate	S.E.	P-value	Estimate	S.E.	P-value
**Glucose Homeostasis Related**						
Glucose (mg/dL)	0.011	0.01	0.23	-0.03	0.06	0.61
Insulin (mIU/L)	0.0024	0.002	0.21	-0.02	0.01	0.17
HOMA-IR	0.0005	0.0004	0.19	-0.001	0.003	0.16
Glycosylated hemoglobin (%)	0.20	0.38	0.60	-1.55	2.85	0.59
**CKD-related**						
Serum Albumin (g/dL)	-0.64	0.35	0.07	-0.84	2.40	0.73
Calcium (mg/dL)	-0.39	0.27	0.15	-4.23	1.84	0.02
eGFR (mL/min/1.73 m^2^; MDRD equation)	-0.001	0.01	0.85	0.02	0.04	0.57
Hemoglobin (gm/dL)	0.17	0.10	0.07	0.26	0.70	0.70
Parathyroid hormone (pg/ml)	0.002	0.004	0.67	0.05	0.03	0.10
Phosphorus (mg/dL)	-0.03	0.07	0.70	-0.81	0.51	0.11
Urine Albumin to Creatinine Ratio (mg/g)	0.001	0.0006	0.046	0.003	0.004	0.45
**Other**						
Aldosterone (ng/dL)	-0.0005	0.001	0.36	0.001	0.004	0.76
Uric Acid (mg/dL)	-0.09	0.07	0.19	-0.76	0.47	0.11

Each row is one model. Negative estimates imply an inverse relationship. HOMA-IR = insulin resistance index

## Discussion

The SPRINT study population provides an extremely well characterized participant cohort in whom additional information derived from measures of PWV and central blood pressure were evaluated. These results from a subgroup of the SPRINT study cohort demonstrate that their measures of vascular stiffness are lower than a Grade I Hypertension population for persons age 60 years and older. Furthermore, adjusted linear regression analyses for PWV and C-SBP results identified a marginally statistically significant and modest association between PWV and UACR.

SPRINT was designed to recruit “a diverse population with hypertension and existing cardiovascular disease, existing chronic kidney disease, or an elevated estimated risk for cardiovascular disease based on age and other risk factors.”[13] As shown in Table 1, the SPRINT PWV ancillary study cohort differed from the overall SPRINT cohort in several characteristics–the proportion of patients with CKD was higher and the proportion without CKD but with higher Framingham risk scores was lower–reflecting some differences in demographics at the participating centers. Many studies and recent meta-analyses provide evidence that vascular stiffness as assessed by PWV is an indicator of CVD risk that is independent of peripheral BP. The PWV results from this SPRINT cohort provide additional evidence that the cohort is characterized by high CVD risk. When compared to a reference population with Grade I hypertension, PWV results from SPRINT are similar for the age group 50–59 years, and lower for those age 60 years and older. Several factors may account for these findings. Although the reference population cited is the largest cohort for whom data are available by age decade, its Grade I hypertension group included a range of blood pressure between 140/90 and 160/100 mm Hg and consequently has higher average blood pressure than the SPRINT PWV cohort whose average BP at study entry was 140.5/75.1 mm Hg. In addition, Grade I hypertension participants in the reference population study were untreated whereas approximately 90% of SPRINT participants were receiving antihypertensive treatment. Third, the reference population was recruited from 8 European countries and racial/ethnic differences could influence the comparison. Fourth, it should be noted that the path length methodology used in the reference value population standardized its PWV calculations to be 80% of the direct carotid to femoral distance. This approach has been shown to result in lower PWV values relative the subtracted distance method [18]

With respect to demographics, there was only one significant finding: C-SBP was higher in women than men in SPRINT, in parallel with the sex difference in brachial, peripheral BP. The generalized linear model relating C-SBP to BMI was of borderline statistical significance, but in the same direction identified in the unadjusted correlation. The unexpected lack of (for PWV) and inverse (for C-SBP) relation with BMI may be explained by the selection criteria that were used for SPRINT as a whole, where persons with diabetes were excluded, and for this ancillary study in particular, since participants with higher BMI were more likely to have PWV studies that could not be conducted or that failed to meet quality control parameters.

Based on prior investigations that have identified higher PWV in persons with diabetes [19–21] or with higher levels of insulin resistance in the absence of diabetes [22, 23], we had hypothesized that markers of impaired glucose homeostasis or insulin resistance would be associated with PWV and/or C-SBP. The linear regression model results adjusted for age, sex, and race/ethnicity shown in Table 2 failed to support this hypothesis. Moreover, with the exception of only the relatively weak relations between PWV and UACR, there was a general lack of association between PWV or C-SBP and the markers related to CKD, aldosterone, or uric acid.

Several limitations inherent to this study’s design merit consideration. The SPRINT cohort is not a population-based sample. The randomized clinical trial participant population had several exclusions–notably diabetes–and may not be generalizable to a general hypertensive population. The age distribution of the population–age 50 years to 90s –may represent another challenge to its generalizability.

## Conclusions

In a subset of SPRINT participants, PWV was significantly higher than in prior studies of normotensive persons, as expected. For older age groups in the SPRINT cohort (age > 60 years), PWV was lower than a reference comparison population of hypertensive individuals. There were no compelling associations noted between PWV or C-SBP and markers of glucose homeostasis or CKD.

## Supporting information

S1 Table Baseline values for biochemical parameters used in the linear regression analyses reported in Table 3. (DOCX)
