# Supplementary material for: Pulse wave velocity and central aortic pressure in systolic blood pressure intervention trial participants
Source: PLoS One. 2018 Sep 26;13(9):e0203305. doi: 10.1371/journal.pone.0203305 (PMC6157848; doi:10.1371/journal.pone.0203305)
Supplement: S1 Table — (DOCX) [file pone.0203305.s001.docx]

**S1 Table. Baseline values for biochemical parameters used in the linear regression analyses reported in Table 3.**

| **Variable** | **N** | **Mean** | **Std Dev** | **Median** | **25th Pctl** | **75th Pctl** | **Minimum** | **Maximum** |
| --- | --- | --- | --- | --- | --- | --- | --- | --- |
| \| **Serum Albumin (g/dL)** \| \| --- \| \| **Calcium (mg/dL)** \| \| **Insulin (mIU/L)** \| \| **Phosphorus (mg/dL)** \| \| **Uric Acid (mg/dL)** \| \| **Glycosylated hemoglobin (%)** \| \| **Hemoglobin (gm/dL)** \| \| **Parathyroid hormone (pg/ml)** \| \| **Glucose (mg/dL)** \| \| **HOMA-IR; insulin resistance index** \| \| **eGFR (mL/min/1.73 m²** \| \| **Urine microalbumin (mg/L)** \| \| **Aldosterone (ng/dL)** \| \| **Urine Albumin to Creatinine Ratio (mg/g)** \| | \| 644 \| \| --- \| \| 644 \| \| 643 \| \| 644 \| \| 644 \| \| 292 \| \| 293 \| \| 599 \| \| 651 \| \| 643 \| \| 650 \| \| 642 \| \| 612 \| \| 619 \| | \| 4.1 \| \| --- \| \| 9.6 \| \| 63.9 \| \| 3.8 \| \| 6.3 \| \| 5.6 \| \| 13.8 \| \| 45.5 \| \| 95.6 \| \| 280.9 \| \| 67.5 \| \| 51.7 \| \| 144.7 \| \| 53.2 \| | \| 0.3 \| \| --- \| \| 0.4 \| \| 55.6 \| \| 1.4 \| \| 1.7 \| \| 0.4 \| \| 1.6 \| \| 26.8 \| \| 11.4 \| \| 283.0 \| \| 20.1 \| \| 173.8 \| \| 208.1 \| \| 166.0 \| | \| 4.1 \| \| --- \| \| 9.5 \| \| 50.0 \| \| 3.6 \| \| 6.2 \| \| 5.7 \| \| 13.8 \| \| 38.6 \| \| 94.0 \| \| 212.8 \| \| 67.5 \| \| 10.0 \| \| 74.5 \| \| 10.9 \| | \| 3.9 \| \| --- \| \| 9.3 \| \| 32.0 \| \| 3.2 \| \| 5.2 \| \| 5.4 \| \| 12.9 \| \| 30.6 \| \| 88.0 \| \| 133.8 \| \| 53.9 \| \| 5.0 \| \| 54.2 \| \| 6.4 \| | \| 4.3 \| \| --- \| \| 9.8 \| \| 79.0 \| \| 4.0 \| \| 7.2 \| \| 5.9 \| \| 14.8 \| \| 50.9 \| \| 102.0 \| \| 348.4 \| \| 81.3 \| \| 26.0 \| \| 117.5 \| \| 26.3 \| | \| 3.2 \| \| --- \| \| 8.2 \| \| 3.0 \| \| 1.7 \| \| 1.8 \| \| 4.4 \| \| 7.6 \| \| 11.9 \| \| 45.0 \| \| 11.9 \| \| 14.7 \| \| 2.0 \| \| 22.6 \| \| 2.3 \| | \| 5.1 \| \| --- \| \| 11.4 \| \| 677.0 \| \| 22.3 \| \| 13.5 \| \| 6.8 \| \| 19.0 \| \| 289.2 \| \| 160.0 \| \| 3640.8 \| \| 135.1 \| \| 2110.0 \| \| 1000.0 \| \| 1956.8 \| |
